# Acute resveratrol exposure does not impact hemodynamics of the fetal sheep

**DOI:** 10.14814/phy2.15749

**Published:** 2023-06-18

**Authors:** Jack R. T. Darby, Georgia K. Williams, Steven K. S. Cho, Ashley S. Meakin, Stacey L. Holman, Megan Quinn, Michael D. Wiese, Christopher K. Macgowan, Mike Seed, Janna L. Morrison

**Affiliations:** ^1^ Early Origins of Adult Health Research Group UniSA: Clinical and Health Sciences University of South Australia South Australia Adelaide Australia; ^2^ Preclinical, Imaging and Research Laboratories South Australian Health and Medical Research Institute Adelaide South Australia Australia; ^3^ Univeristy of Toronto and The Hospital for Sick Children Toronto Ontario Canada; ^4^ Centre for Pharmaceutical Innovation, UniSA: Clinical and Health Sciences University of South Australia South Australia Adelaide Australia

**Keywords:** cardiovascular, fetal development, fetus, FGR, hemodynamics, IUGR, MRI, PC‐MRI, resveratrol, T_2_ oximetry

## Abstract

Babies born growth restricted are at an increased risk of both poor short‐and long‐term outcomes. Current interventions to improve fetal growth are ineffective and do not lower the lifetime risk of poor health status. Maternal resveratrol (RSV) treatment increases uterine artery blood flow, fetal oxygenation, and fetal weight. However, studies suggest that diets high in polyphenols such as RSV may impair fetal hemodynamics. We aimed to characterize the effect of RSV on fetal hemodynamics to further assess its safety as an intervention strategy. Pregnant ewes underwent magnetic resonance imaging (MRI) scans to measure blood flow and oxygenation within the fetal circulation using phase contrast‐MRI and T_2_ oximetry. Blood flow and oxygenation measures were performed in a basal state and then repeated while the fetus was exposed to RSV. Fetal blood pressure and heart rate were not different between states. RSV did not impact fetal oxygen delivery (DO_2_) or consumption (VO_2_). Blood flow and oxygen delivery throughout the major vessels of the fetal circulation were not different between basal and RSV states. As such, acute exposure of the fetus to RSV does not directly impact fetal hemodynamics. This strengthens the rationale for the use of RSV as an intervention strategy against fetal growth restriction.

## 1 INTRODUCTION

Impaired fetal substrate supply results in fetal growth restriction (FGR; ~10% of all pregnancies), a condition in which the developing fetus does not have a sufficient oxygen and/or nutrient supply to reach its genetically predetermined growth potential (Sharma et al., 2016). While FGR increases the risk of poor short‐term outcomes such as preterm delivery and stillbirth (Bukowski et al., 2014), it also predisposes offspring to the development of chronic disease across the life course (Barker et al., 1989; McMillen & Robinson, 2005). As such, there is great interest in the development of intervention strategies that can restore fetal substrate supply to improve outcomes in these complicated pregnancies.

One candidate for intervention in FGR is the polyphenol resveratrol (RSV). RSV has vasodilator properties through its role in enhancing nitric oxide bioactivity by acting as a potent activator of sirtuin‐1 (SIRT1). It also has direct antioxidant effects (Hannan et al., 2017); a characteristic that would be beneficial in the setting of FGR where the associated fetal hypoxemia is often accompanied by an increased generation of damaging reactive oxygen species that contribute toward fetal programming of later life chronic disease (Alexander et al., 2015; Giussani et al., 2012; Rueda‐Clausen et al., 2012).

We performed a systematic review to better understand both maternal and fetal outcomes from complicated pregnancies where RSV was trialed as an intervention (Darby, Mohd Dollah, et al., 2019). This review identified RSV's ability to improve uterine artery blood flow both in the context of preeclampsia (Poudel et al., 2013) and maternal western diet exposure (Roberts et al., 2014). Importantly, only a handful of studies reported maternal plasma resveratrol concentrations and those that reported the corresponding fetal concentrations did so at only one time point. Given that the timing, severity, and duration of an in utero insult dictates outcomes (Darby, Varcoe, et al., 2020; Morrison, 2008), the same is possible for gestational RSV exposure whereby different ranges of RSV concentrations may yield a variety of outcomes.

Determining the RSV concentrations within the fetal circulation during a maternal RSV intervention is crucial to characterize RSV's potential as an intervention strategy. To address this gap in knowledge, we used the well characterized chronically catheterized pregnant sheep (Morrison et al., 2018). RSV‐loaded implants were placed subcutaneously in the pregnant ewe and fetal catheterization surgery was performed to allow for repetitive fetal blood sampling across gestation. Maternal RSV treatment increased uterine artery blood flow, improved fetal oxygenation status, and increased fetal body weight (Darby, Saini, et al., 2019), with no impact on the fetal hemodynamics (Aujla et al., 2021). However, although we detected stable concentrations of RSV within the maternal circulation, we did not detect RSV in fetal plasma, indicating that, unlike rodents (Bourque et al., 2012), nonhuman primates (Roberts et al., 2014), and likely humans, RSV does not cross the sheep placenta. Thus, the chronically catheterized fetal sheep is a unique model in which to tease apart the direct maternal and indirect fetal effects of RSV exposure from the direct fetal effects of RSV exposure.

While RSV may increase nitric oxide production and thus decrease the vascular tone of the fetal cardiovascular system (Xia et al., 2014), there is evidence that polyphenols such as RSV may inhibit prostaglandin synthesis leading to constriction of the ductus arteriosus (DA; Zielinsky & Busato, 2013). During fetal life, blood oxygenation occurs at the placenta with no gas exchange occurring at the fluid filled lungs. The DA allows the majority of right ventricular cardiac output (RVCO) to bypass the pulmonary circulation and flow into the descending aorta (Mielke & Benda, 2001; Prsa et al., 2014; Rudolph, 1985). Although DA closure is necessary at birth when the site of gas exchange shifts from the placenta to the lungs, premature DA constriction in utero is associated with right ventricular dysfunction, fetal hydrops, fetal mortality, and an increased risk of developing neonatal pulmonary hypertension (Enzensberger et al., 2012; Gewillig et al., 2009). Assessing the hemodynamic consequences of fetal RSV exposure is therefore of utmost importance in establishing the safety of RSV as an intervention strategy during the complicated pregnancy.

We have previously validated the use of phase‐contrast (PC)‐magnetic resonance imaging (MRI) and T_2_ oximetry to measure blood flow and oxygen saturation to then calculate oxygen delivery within the circulations of both the human and sheep fetus (Saini et al., 2020). Moreover, the combination of these two clinically relevant MRI techniques has proven useful in understanding the impact that vasoactive compounds have on fetal hemodynamics (Darby, Schrauben, et al., 2020; Dimasi et al., 2021; Duan et al., 2019; Morrison et al., 2022; Saini et al., 2020). Herein, we aimed to utilize these advanced MRI techniques to determine the direct impact that RSV has on the hemodynamics of the fetal sheep. We hypothesized that acute fetal RSV exposure would have no impact on blood flow through the DA.

## 2 MATERIALS AND METHODS

### 2.1 Ethical considerations

All experimental protocols were reviewed and approved by the Animal Ethics Committee of the South Australian Health and Medical Research Institute (SAHMRI) and abide by the Australian Code of Practice for the Care and Use of Animals for Scientific Purposes developed by the National Health and Medical Research Council. Ewes from the SAHMRI farm (Burra, South Australia) were housed in an indoor facility with a constant ambient temperature of 20–22°C and a 12‐h light/dark cycle. Ewes were housed in individual pens in view of other sheep and had ad libitum access to food and water. All investigators understood the ethical principles outlined in Grundy (2015) and the principles of the 3Rs, specifically the reduction of the use of animals in research (Russell & Burch, 1959).

### 2.2 Fetal catheterization surgery

At 116–117 days gestational age (GA; term = ~150 days), Merino ewes carrying singleton fetuses (*n* = 6; 5:1 female:male) underwent surgery under aseptic conditions as previously described (Edwards et al., 1999; Morrison et al., 2001). Anesthesia was induced with intravenous diazepam (0.3 mg/kg) and ketamine (5 mg/kg) and then maintained with isoflurane (1.5%–2.5% in 100% oxygen). Vascular catheters were implanted into the maternal jugular vein, fetal femoral vein, femoral artery, and the amniotic cavity as previously described (Edwards et al., 1999; Morrison et al., 2001). Ewes received an intramuscular injection of antibiotics (3.5 mL of Duplocillin [150 mg/mL procaine penicillin and 112.5 mg/mL benzathine penicillin; Norbrook Laboratories Ltd.]) and 2 mL of 125 mg/mL Dihydrostreptomycin (Sigma‐Aldrich) at surgery and for 3 days following surgery. Fetuses received an intramuscular injection of 1 mL of Duplocillin (150 mg/mL procaine penicillin and 112.5 mg/mL benzathine penicillin) and 1 mL of 125 mg/mL Dihydrostreptomycin during surgery. All ewes received an analgesic, meloxicam (0.5 mg/kg, subcutaneously) on the day before surgery and 24 h later (Varcoe et al., 2019). Each fetus received antibiotics (500 mg; sodium ampicillin; Commonwealth Serum Laboratories) intra‐amniotically for 4 days post‐surgery.

### 2.3 Experimental protocol

Pregnant ewes underwent MRI scans between 120 and 124 days GA (e.g., mid‐third trimester) after ~16 h of fasting. General anesthesia was induced in the ewe as described for surgery above. The ewe was then positioned on its left side for the duration of the scan and ventilated to ensure normal fetal oxygenation levels (respiratory rate 16–18; ~1 L O_2_ and 5 L air). Maternal heart rate and arterial oxygen saturation were measured using an MRI compatible SaO_2_/heart rate monitor (Nonin Medical, Inc.). The sensor was placed on the pregnant ewes' teat and measurements were continuously recorded using LabChart 7 (Darby, Saini, et al., 2019; Duan et al., 2019).

The fetal femoral artery and amniotic catheters were connected to displacement transducers, a quad‐bridge amplifier and a data acquisition unit (PowerLab; ADInstruments) to record fetal blood pressure (corrected for amniotic pressure). All data were sampled at a rate of 1000 Hz, digitized and recorded using LabChart 7 (ADInstruments). The resulting blood pressure signal acted as a real‐time external cardiac trigger for fetal MRI scanning (Duan et al., 2017, 2019; Schrauben et al., 2019).

Imaging was performed on a 3 T clinical MRI system (MAGNETOM Skyra; Siemens Healthineers). Fetal blood flow measurements and oxygen saturations were determined by PC‐MRI and T_2_ MRI oximetry as previously described (Darby, Schrauben, et al., 2020; Duan et al., 2019; Saini et al., 2020). Measurements were taken in a basal state after a vehicle bolus (50 μL DMSO in 3 mL saline) and then after a bolus dose of RSV (50 μg; Sigma‐Aldrich) into the fetal femoral vein. This dose was based on pilot data and set to target fetal RSV plasma concentrations as previously described in fetal nonhuman primates after maternal dietary RSV supplementation (Roberts et al., 2014). MRI acquisition during the RSV state began at 15 min after the RSV bolus was given and lasted ~45 min.

### 2.4 Determination of blood flow within the fetal circulation

The fetal femoral arterial pressure waveform was used to generate a cardiac trigger for MRI (Duan et al., 2017; Schrauben et al., 2019). Two‐dimensional cine PC imaging was performed to measure blood flow within the fetal circulation with corresponding vessel appropriate velocity encoding (VENC). PC‐MRI acquisitions were completed for the ascending aorta (AAo; 150 cm/s), main pulmonary artery (MPA; 150 cm/s), descending aorta (DAo; 150 cm/s), superior vena cava (SVC; 100 cm/s), DA (150 cm/s), left and right pulmonary arteries (LPA/RPA; 80 cm/s), combined carotid arteries (100 cm/s), umbilical vein (UV; 100 cm/s), and ductus venosus (100 cm/s) using the following parameters: flip angle: 30°, repetition time: 7 ms, echo time: 3.18 ms, field of view: 240 mm, in‐plane resolution: 1.0 × 1.0 mm^2^, slice thickness: 5.0 mm, number of signal averages: 3, and views per segment: 2 according to our previously published technique (Cho et al., 2020; Dimasi et al., 2021; Duan et al., 2019). With ~15 acquired phases in the cardiac cycle, these parameters achieve a temporal resolution of ~28 ms. The typical acquisition time for each vessel was ~2 min. PC cine images were acquired in the short axis plane of the vessels of interest, which were prescribed using two perpendicular long axis views of each vessel. Pulmonary blood flow (PBF) was determined as the sum of blood flow in the LPA and RPA. RVCO was determined as the sum of DA and pulmonary blood flows. Left ventricular output (LVCO) was determined as equal to AAo blood flow and did not include coronary blood flow.

### 2.5 Determination of oxygen saturation within the fetal circulation

Due to the paramagnetic properties of deoxyhemoglobin, the T_2_ relaxation time of blood is related to the oxygen saturation of blood (Christen et al., 2013). Vessel T_2_ oximetry was performed using a T_2_‐prepared pulse sequence with a balanced steady‐state free precession acquisition (Myomaps; Siemens) (Saini et al., 2020; Sun et al., 2015; Xu et al., 2020; Zhu et al., 2016). In‐plane resolution was 1.3 × 1.3 mm. MRI acquisition parameters over all subjects and vessels were: repetition time = 4.2 ms, echo time = 2.1 ms, flip angle = 70°, slice thickness = 6 mm, T_2_ preparation times = [32, 64, 96, 128, 160, 192] ms, and acquisition time = ~50 s. A non‐rigid motion correction algorithm was applied to compensate for slight in‐plane fetal movement (co‐registration) (Giri et al., 2012).

The T_2_ relaxation time for each vessel of interest was analyzed using CVI^42^ (Circle Cardiovascular Imaging). The regions‐of‐interest were manually adjusted for each image slice to cover the central 60% of the vessel of interest (UV, AAo, DAo, MPA, and SVC) (Stainsby & Wright, 1998). Oxygen saturation was then calculated from T_2_ relaxation time using the T_2_‐oxygen saturation relationship for sheep blood as previously described (Saini et al., 2020).

### 2.6 Determination of oxygen delivery and consumption

Blood flow and T_2_‐derived oxygen saturations were combined to calculate overall fetal oxygen delivery (DO_2_), fetal oxygen consumption (VO_2_), cerebral DO_2_, cerebral VO_2_, and pulmonary DO_2_ using the following equations:

1. Fetal DO_2_:

$$DO_{2}=1.36\times \left[\mathrm{Hb}\right]\times Y_{\mathrm{UV}}\times Q_{\mathrm{UV}}$$

2 Cerebral DO_2_:

$${\mathrm{DO}}_{2}=1.36\times \left[\mathrm{Hb}\right]\times Y_{\mathrm{AAo}}\times Q_{\mathrm{CCa}}$$

3 Fetal VO_2_:

$${\mathrm{VO}}_{2}=1.36\times \left[\mathrm{Hb}\right]\times \left(Y_{\mathrm{UV}}-Y_{\mathrm{DAo}}\right)\times Q_{\mathrm{UV}}$$

4 Cerebral VO_2_:

$${\mathrm{VO}}_{2}=1.36\times \left[\mathrm{Hb}\right]\times \left(Y_{\mathrm{AAo}}-Y_{\mathrm{SVC}}\right)\times Q_{\mathrm{CCa}}$$

*Q*_UV_ represents the measured umbilical vein blood flow; *Q*_CCa_ represents the combined blood flow of the carotid arteries; *Q*_PBF_ represents the combined blood flow in the left and right pulmonary arteries; [Hb] represents the mean fetal hemoglobin concentration during MRI scan; 1.36 is the amount of oxygen (mL at one atmosphere) bound per gram of hemoglobin; *Y*_UV_ represents the oxygen saturation of UV blood; *Y*_DAo_ represents the oxygen saturation of the DAo blood, *Y*_AAo_ represents the oxygen saturation of AAo blood, and *Y*_SVC_ represents the oxygen saturation of the SVC.

### 2.7 Blood sampling and fetal blood gas measurements

After fetal surgery, fetal arterial blood samples (0.5 mL) were collected daily to monitor fetal health by measuring the partial pressure of oxygen (PaO_2_), partial pressure of carbon dioxide (PaCO_2_), oxygen saturation (SaO_2_), pH, hemoglobin (Hb), hematocrit (Hct), base excess, and lactate concentrations, and the temperature was corrected to 39°C for sheep blood with a RAPIDPOINT 500 (Siemens Healthineers). During the MRI scan, arterial samples (0.5 mL) for fetal blood gas analysis were taken at the beginning and end of each state and blood samples (3 mL) were collected at 15, 45, and 60 min post RSV administration for subsequent RSV plasma concentration analysis.

### 2.8 Determination of fetal resveratrol plasma concentrations

Fetal resveratrol plasma concentrations were determined using liquid chromatography–tandem mass spectrometry as per our previously published protocol (Darby, Saini, et al., 2019). Briefly, 100 μL fetal plasma was incubated with 10 μL of 100 ng/mL stable isotope RSV (Resveratrol‐13C6; Toronto Research Chemicals) as an internal standard, 80 μL 0.1 M sodium acetate buffer (pH 5.0) and 20 μL β‐glucuronidase type HP‐2 (50,000 units/L in 0.1 M sodium acetate buffer [pH 5.0]; Sigma, # G7017) in a water bath for 4 h at 37°C. After the incubation period, 1 mL of 100% acetonitrile (Honeywell; #494445) was added and vortexed for a total of 5 min. Samples were centrifuged at 11,000 *g* for 10 min and the supernatant was removed and evaporate, and the residue was reconstituted in 30% methanol. 25 μL of sample was injected on a Kinetex 1.7 μm F5 LC Column (150 × 2.1 mm; Phenomenex). Mobile phase A was 0.5 mM ammonium fluoride in 5% methanol, and mobile phase B was 0.5 mM ammonium fluoride in 95% methanol. The flow rate was 0.3 mL/min and mobile phase B was initially 30% and increased linearly to 100% at 3.5 min and then held to 4.25 min, after which it was reduced to 30% at 4.5 min and finally held at 30% B for 6.5 min prior to injection of the next sample. RSV and the stable isotope RSV were detected using a SCIEX 4500 Triple‐Quad (SCIEX), which was operated in negative ion mode. Multiple reaction monitoring transitions for RSV and the stable isotope RSV are previously described (Darby, Saini, et al., 2019). Standards were prepared by spiking blank sheep plasma with seven different concentrations of RSV (0.5–40 ng/mL) and were subjected to the same process as above. The ratio of the area of the RSV to internal standard peaks were used to generate standard curves and the concentration of RSV in plasma samples were determined by linear regression.

### 2.9 Postmortem

At 123–124 days GA pregnant ewes were humanely killed with an overdose of sodium pentobarbitone (150 mg/kg; Virbac) and the fetus was delivered via hysterotomy and weighed. The fetal body and brain were weighed for blood flow normalization.

### 2.10 Statistical analysis

To determine the effect of RSV on fetal blood flow, oxygen delivery, and consumption, a two‐tailed paired *t*‐test was used (GraphPad Prism version 8; GraphPad Software). The impact of RSV on fetal blood pressure and heart rate was determined using a repeated measures one‐way ANOVA with time (analyzed as 5 min averages every 5 min) as the repeated measure. Based on our previous work using MRI to measure the impact of vasoactive agents on fetal hemodynamics (Darby, Schrauben, et al., 2020), a sample size of *n* = 6 is sufficient to achieve 80% power for a paired analysis. Data are presented as mean ± SD and a probability of 5% (*p* < 0.05) was considered significant for all analyses.

## 3 RESULTS

### 3.1 Fetal plasma RSV concentrations, blood gas, pH, Hb, Hct, and lactate measures

**FIGURE 1:**
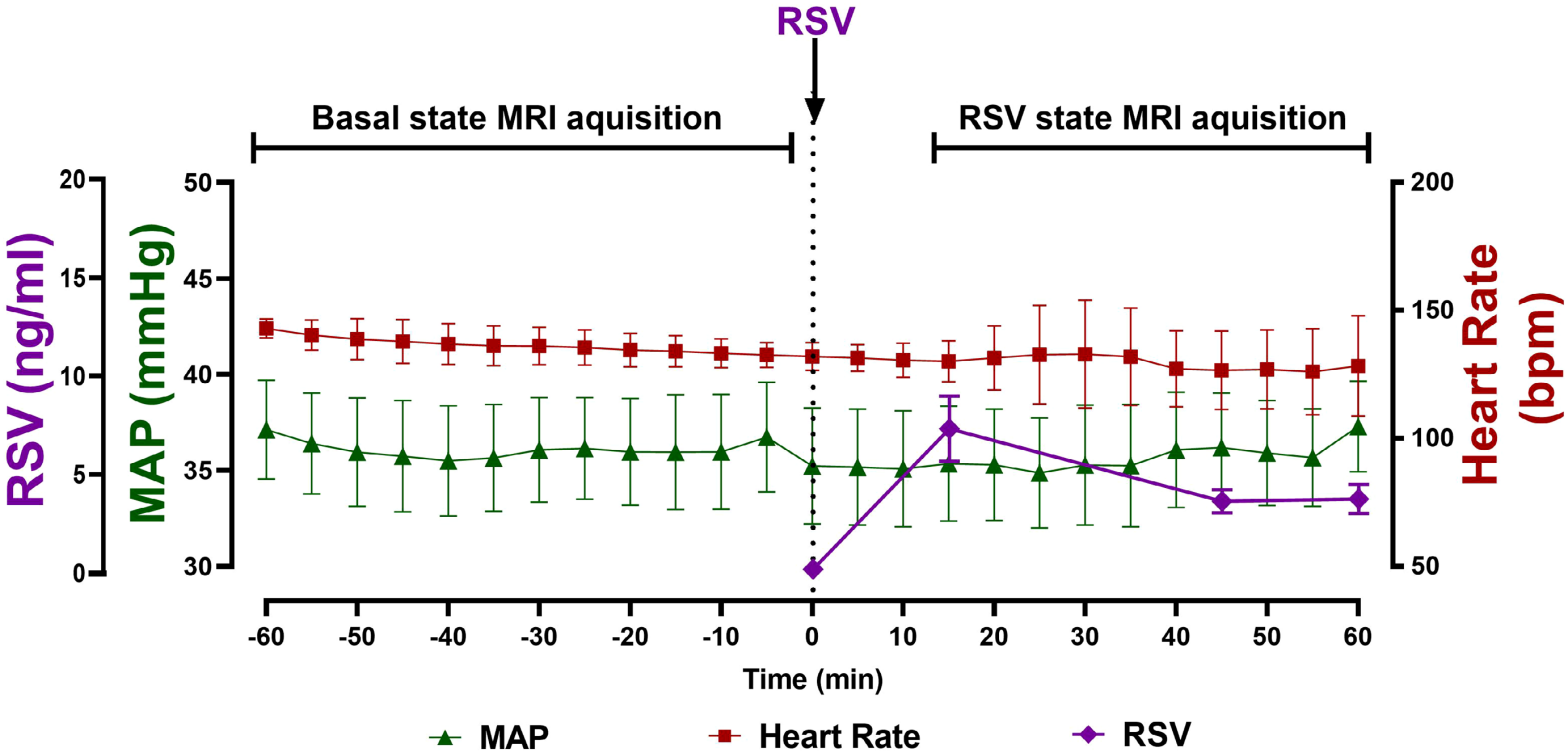
Fetal MAP (triangles), HR (squares), and plasma RSV concentrations (diamonds) across the basal and RSV MRI acquisition windows (*n* = 6). Data analyzed by a repeated measures one‐way ANOVA with a Bonferroni correction for multiple comparisons. Data presented as mean ± SD. *p* ≤ 0.05. HR, heart rate; MAP, mean arterial pressure; RSV, resveratrol.

Fetal weight was comparable between fetuses (3.090 ± 0.137; mean ± SD). Following fetal RSV administration, fetal plasma RSV plasma concentrations peaked at 7.13 ± 4.17 ng/mL (mean ± SD) and then remained within the desired range (~5 ng/mL; range = 1.93–5.85 ng/mL) for the duration of the RSV MRI acquisition window (Figure 1).

Fetal PaCO_2_, SaO_2_, Hb, and Hct remained stable from prior to anesthesia and throughout both the basal and RSV acquisition windows. There was no difference in fetal PaO_2_ between basal and RSV states. However, fetal PaO_2_ was significantly higher (albeit not outside the physiological range for Merino fetal sheep at this GA) during both MRI states than prior to anesthesia (Table 1). There was no difference in fetal pH and lactate between basal and RSV states; however, fetal pH was significantly lower during both MRI states than prior to anesthesia and fetal lactate was significantly higher during both MRI states than prior to anesthesia (Table 1).

**TABLE 1:** Fetal blood gases, Hb, Hct, and lactate values prior to anesthesia for MRI and during basal and RSV MRI acquisition states.

	Pre‐anesthesia (*n* = 6)	Basal state (*n* = 6)	RSV state (*n* = 6)	*p*‐Value
PaO_2_ (mmHg)	17.6 ± 1.3^a^	21.9 ± 2.9^b^	20.5 ± 3.6^ab^	0.0209
PaCO_2_ (mmHg)	49.8 ± 6.3	52.3 ± 6.6	55.4 ± 10.8	0.0834
pH	7.374 ± 0.021^a^	7.308 ± 0.032^b^	7.307 ± 0.027^b^	0.0027
SaO_2_ (%)	54.6 ± 7.0	60.8 ± 11.8	57.0 ± 10.5	0.4256
Hb (g/L)	100.2 ± 7.9	96.9 ± 13.9	99.4 ± 13.3	0.4733
Hct (%)	29.7 ± 2.3	28.6 ± 4.2	29.2 ± 3.9	0.4432
Lactate (mmol/L)	1.22 ± 0.17^a^	2.14 ± 0.40^b^	2.72 ± 0.51^b^	0.0028

*Note*: Values are mean ± SD. Ewes were anesthetized and lying on their left side during MRI. Data analyzed by repeated measures one‐way ANOVA with Bonferroni's correction for multiple comparisons. Superscript alphabetical letters indicate significant differences between states (*p* < 0.05) such that values with different alphabetical letters are statistically different from each other and values with the same alphabetical letters are not different.

Abbreviations: Hb, hemoglobin; Hct, hematocrit; MRI, magnetic resonance imaging; PaCO_2_, partial pressure of carbon dioxide; PaO_2_, partial pressure of oxygen; SaO_2_, oxygen saturation; RSV, resveratrol.

### 3.2 Impact of RSV on fetal blood pressure and heart rate

There was no impact of RSV exposure on fetal MAP or heart rate compared to the basal state (Figure 1).

### 3.3 Impact of RSV on umbilical vein blood flow, ductus venous shunting, fetal oxygen delivery, and consumption

**FIGURE 2:**
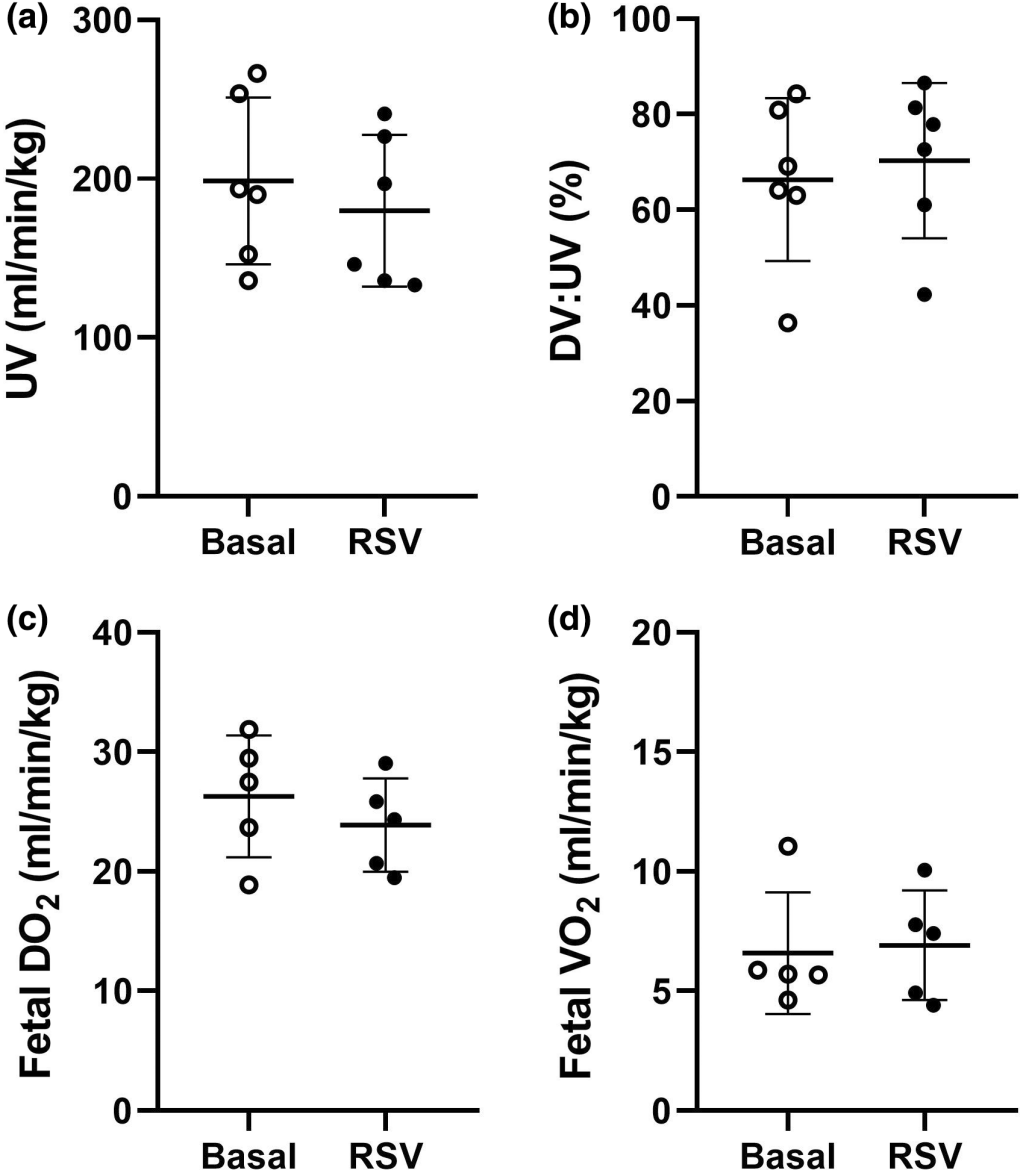
Umbilical vein blood flow (a), ductus venous shunting (b), fetal oxygen delivery (c), and fetal oxygen consumption (d) during basal (unfilled circles) and RSV (filled circles) MRI acquisition windows. One animal excluded from fetal DO_2_ and VO_2_ analysis due to unusable T_2_ prescription of the UV (fetal movement). Data analyzed by a paired *t*‐test. *p* ≤ 0.05. DV, ductus venosus; RSV, resveratrol; UV, umbilical vein.

Fetal RSV exposure did not impact umbilical vein blood flow (Figure 2a), the proportion of oxygen‐rich blood shunted through the ductus venosus (Figure 2b), overall fetal DO_2_ (Figure 2c), or fetal VO_2_ (Figure 2d) compared to the basal state.

### 3.4 Impact of RSV on blood flow within the fetal circulation

**FIGURE 3:**
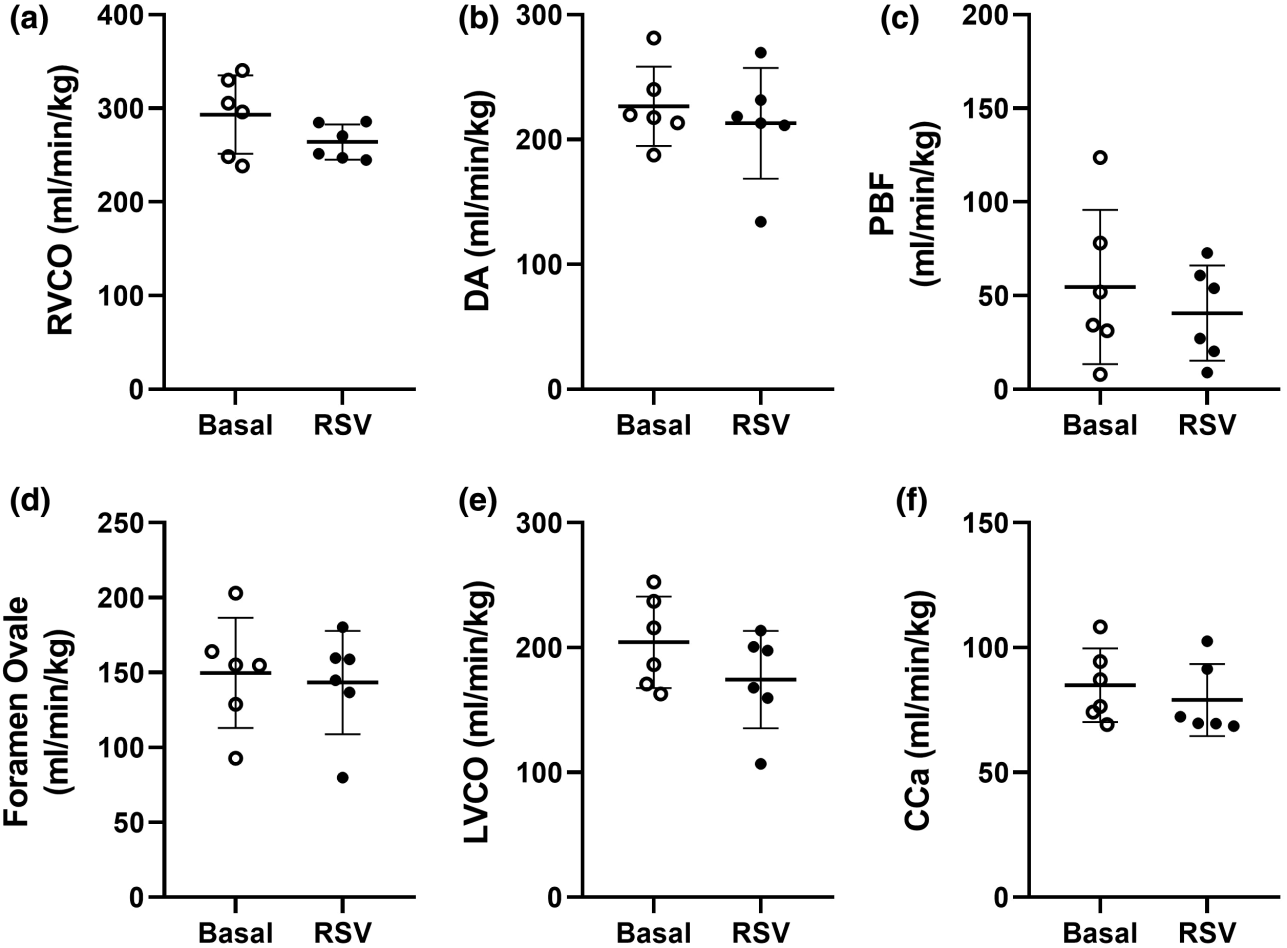
Effect of fetal RSV exposure on right ventricular cardiac output [(a) RVCO], blood flow through the ductus arteriosus [(b) DA], pulmonary blood flow [(c) PBF], blood flow through the foramen ovale [(d) FO], left ventricular cardiac output [(e) LVCO], and combined carotid artery blood flow (f). Data presented as individual data points (*n* = 6; basal state, unfilled circles; RSV state, filled black circles). Data analyzed by a paired *t*‐test. *p* ≤ 0.05. RSV, resveratrol.

Fetal RSV exposure did not impact RVCO (Figure 3a), pulmonary blood flow (Figure 3c) or blood flow through the DA (Figure 3b). In addition, there was no impact of fetal RSV exposure on blood flow from the right to the left side of the heart through the foramen ovale (Figure 3d), LVCO (Figure 3e), or blood flow to the brain through the carotid arteries (Figure 3f).

### 3.5 Effect of RSV on cerebral DO_2_ , cerebral VO_2_ and oxygen extraction

**FIGURE 4:**
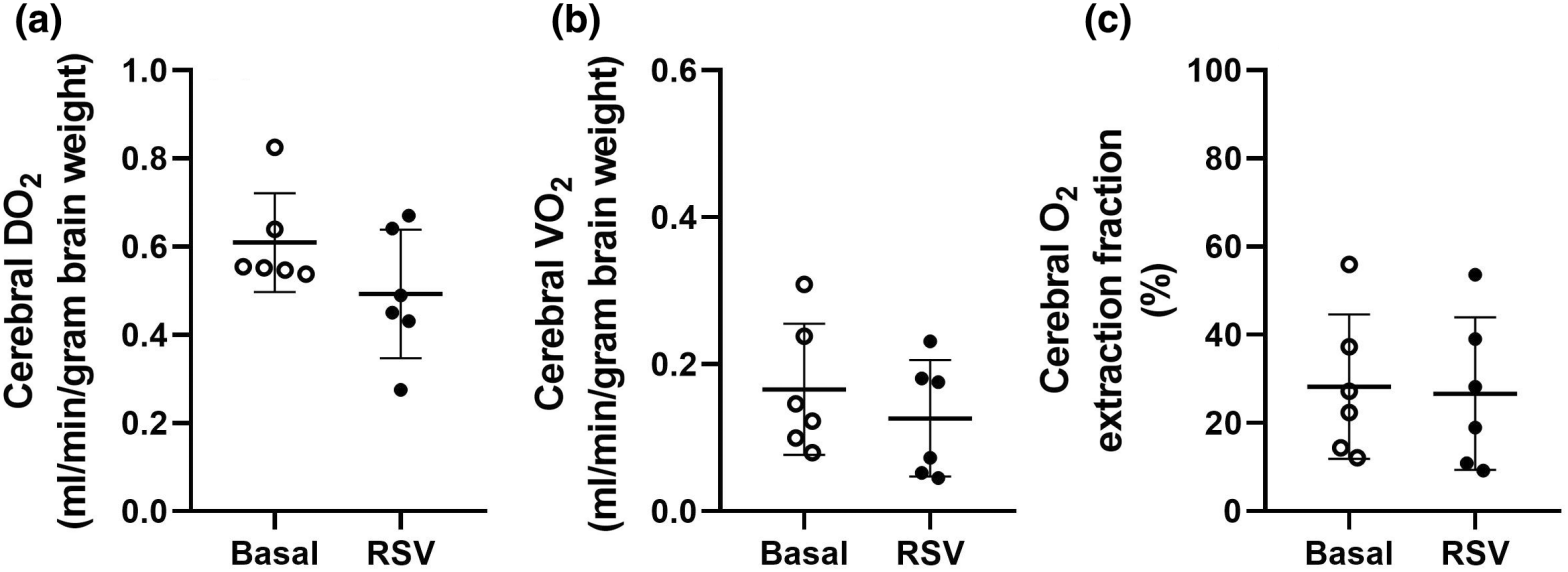
Fetal cerebral DO_2_ (a), cerebral VO_2_ (b), and cerebral oxygen extraction (c) during basal (unfilled circles) and RSV (filled circles) MRI acquisition windows. Data analyzed by a paired *t*‐test. *p* ≤ 0.05. MRI, magnetic resonance imaging; RSV, resveratrol.

Fetal RSV exposure did not impact cerebral DO_2_ (Figure 4a), cerebral VO_2_ (Figure 4b), or cerebral oxygen extraction fraction (Figure 4c).

### 3.6 Effect of RSV on combined ventricular output and blood flow distribution

**FIGURE 5:**
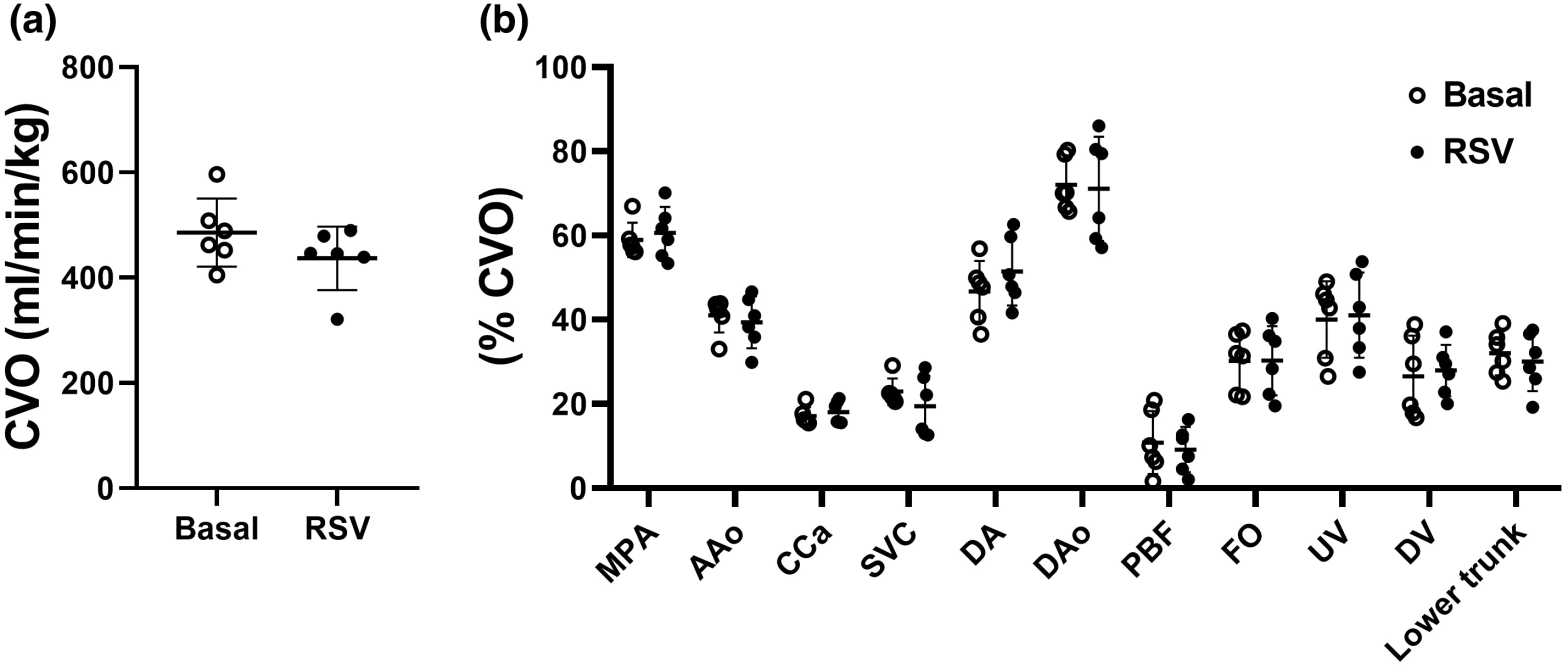
Effect of RSV exposure on combined ventricular output [(a) CVO] and blood flow distribution within the fetal circulation (b). Data presented as individual data points (*n* = 6; basal state, unfilled circles; RSV state, filled black circles). Data analyzed by a paired *t* test. *p* ≤ 0.05. AAo, ascending aorta; CCa, combined carotid arteries; DA, ductus arteriosus; DAo, descending aorta; DV, ductus venosus; FO, foramen ovale; MPA, main pulmonary artery; PBF, pulmonary blood flow; RSV, resveratrol; SVC, superior vena cava; UV, umbilical vein.

Combined ventricular output (CVO) was not changed by fetal RSV exposure (Figure 5a). Blood flow distribution (normalized to CVO) throughout the fetal circulation was not changed by fetal RSV exposure (Figure 5b).

## 4 DISCUSSION

Maternal RSV treatment has shown great potential as an intervention strategy for FGR in that it increases uterine artery blood flow, improves fetal oxygenation, and increases fetal body weight (Bourque et al., 2012; Darby, Mohd Dollah, et al., 2019; Darby, Saini, et al., 2019; Lacerda et al., 2023; Poudel et al., 2013; Roberts et al., 2014). However, as a polyphenol there has been concern that through its inhibition of prostaglandin synthesis, RSV may act directly on the fetal vasculature to constrict and close the DA (Zielinsky & Busato, 2013). In such a scenario, there may be adverse fetal cardiovascular complications including right ventricular dilatation, pulmonary volume overload, and tricuspid regurgitation (Bakas et al., 2020; Gewillig et al., 2009). Herein we utilized clinically relevant MRI techniques in combination with the well characterized chronically catheterized pregnant sheep to determine the direct impact that acute RSV exposure has on fetal hemodynamics.

The DA encompasses a thick circumferential muscle layer that allows for effective constriction, ideally at or around the time of birth, when there is a drop in prostaglandins. However, anti‐inflammatory mediators such as indomethacin and ibuprofen can cause DA closure through their inhibition of prostaglandin synthesis. For this reason, both are used clinically to treat patent DA in the neonatal period (Ohlsson et al., 2015; Poon, 2007; Thomas et al., 2005). In line with this, the majority of in utero DA closure cases are attributed to maternal ingestion of these drugs. However, there remains a substantial proportion of premature DA closure cases with no definitive etiology. One possible cause of these cases may be maternal ingestion of a diet rich in polyphenols with subsequent fetal exposure. Indeed, polyphenols can inhibit prostaglandin synthesis and may thus have a similar impact on the DA as either ibuprofen or indomethacin (Yoon & Baek, 2005).

Studies in humans have provided substantial evidence that maternal dietary intake of polyphenols is associated with DA constriction and impaired DA flow dynamics (Vian et al., 2018; Zielinsky et al., 2010, 2013; Zielinsky, Piccoli Jr., et al., 2012). Notably, these studies did not report or refer to the exact polyphenol breakdown of the participants' diets. This is important as the term polyphenol encompasses more than 8000 plant compounds with unique structures that are broadly classified as either phenolic acids, flavonoids/flavanols (e.g., cacao, green tea), stilbenes (e.g., RSV), or lignans (Pandey & Rizvi, 2009). To the best of our knowledge, the present study is the first to investigate the impact that a purified form of RSV directly has on fetal DA dynamics. Unlike the aforementioned human studies, we found that fetal RSV exposure had no impact on blood flow through the DA, indicating that RSV may not be the polyphenol responsible DA closure in utero. This notion is supported by preclinical studies that have specifically shown the flavonoids within green tea (Zielinsky, Manica, et al., 2012) and cacao (Zielinsky et al., 2021) are capable of causing premature DA closure and have also demonstrated that alongside prostaglandin inhibition an impairment to fetal NO concentrations by polyphenols may be a key contributor (Bubols et al., 2014). Although, RSV has been shown to inhibit the arachidonic acid pathway and thus the formation of the prostaglandins that aid in keeping the DA patent (Meng et al., 2021), RSV through its potent activation of SIRT‐1 is also capable of enhancing NO synthesis (Li & Forstermann, 2009; Milne & Denu, 2008). As such, it is possible that the multifunctional nature of RSV has resulted in the activation of two pathways that have nullified each other's actions on the DA.

Although the literature on premature DA closure following polyphenol exposure directed our focus toward the assessment of DA blood flow, given the vasoactive potential of RSV, a key strength of the present study was our ability to perform a comprehensive analysis on the impact of fetal RSV exposure on blood flow and oxygen delivery throughout the entirety of the fetal circulation. In the context of developing an intervention strategy for use during the complicated pregnancy, the lack of hemodynamic changes that we have found in response to fetal RSV exposure is encouraging as it suggests that RSV at this dose and duration of exposure does not interfere with normal fetal hemodynamics. Importantly, the fetal RSV plasma concentrations under which our hemodynamic assessments were made were similar to those measured in fetal nonhuman primates after a maternal dietary RSV supplementation that increased uterine artery blood flow (Roberts et al., 2014). This implies that the corresponding concentrations of fetal RSV exposure from those maternal concentrations required to have a beneficial impact on utero‐placental physiology are hemodynamically safe for the developing fetus.

Although we have shown that fetal RSV exposure does not impact fetal hemodynamics, the duration of RSV exposure used in the present study was acute (~60 min). Should RSV be used clinically as an intervention for FGR, the developing fetus would be chronically exposed to RSV (weeks). Certainly, different durations and gestational timing of RSV exposure have yielded different outcomes across a range of species and pathologies (Darby, Mohd Dollah, et al., 2019; Lacerda et al., 2023). Indeed, RSV's influence on both the activity of eNOS and the prostaglandin synthesis pathway appears to be dependent on the duration of RSV exposure (Takahashi et al., 2000; Wallerath et al., 2002).This raises the question of the impact that a longer more relevant duration of RSV exposure may have on vascular tone, DA patency, and overall fetal hemodynamics. Furthermore, chronic resveratrol exposure has been shown to impact pancreatic development (Roberts et al., 2014) and thus longer term studies would not be restricted to hemodynamic assessments but instead be well placed to further investigate the direct role that resveratrol plays in this pathology as well as development of other fetal organs including the liver, brain, and heart.

Herein, we utilized a combination of advanced MRI techniques to show that not only is the blood flow through the DA not impacted but that normal fetal hemodynamics are preserved during fetal exposure to relevant fetal RSV concentrations. Future studies are warranted to ensure that a more relevant chronic fetal RSV exposure at these same concentrations remains hemodynamically safe for the developing fetus.

## AUTHOR CONTRIBUTIONS

Conception or design of the work: Jack R. T. Darby, Christopher K. Macgowan, Mike Seed, and Janna L. Morrison. Acquisition or analysis or interpretation of data for the work: Jack R. T. Darby, Georgia K. Williams, Steven K. S. Cho, Ashley S. Meakin, Stacey L. Holman, Megan Quinn, Michael D. Wiese, Christopher K. Macgowan, Mike Seed, and Janna L. Morrison. Drafting the work or revising it critically for important intellectual content: Jack R. T. Darby, BSS, Stacey L. Holman, Christopher K. Macgowan, Mike Seed, and Janna L. Morrison. Final approval of the version to be published and agreement to be accountable for all aspects of the work: All.

## FUNDING INFORMATION

The work was funded by an ARC Discovery Project (DP190102263 to JLM, CKM, MS) and an ARC Future Fellowship (Level 3; FT170100431) to JLM.

## CONFLICT OF INTEREST STATEMENT

The authors have no conflicts of interest.

## ETHICS STATEMENT

All experimental protocols were reviewed and approved by the Animal Ethics Committee of the South Australian Health and Medical Research Institute (SAHMRI) and abide by the Australian Code of Practice for the Care and Use of Animals for Scientific Purposes developed by the National Health and Medical Research Council.

## Data Availability

The data that support the findings of this study are available from the corresponding author upon reasonable request.
